# Dietary Antioxidant Quality Score (DAQS), serum lipids, markers of glucose homeostasis, blood pressure and anthropometric features among apparently metabolically healthy obese adults in two metropolises of Iran (Tabriz and Tehran): a cross-sectional study

**DOI:** 10.1186/s12902-023-01392-5

**Published:** 2023-07-21

**Authors:** Negin Nikrad, Amir Shakarami, Ayda Zahiri Tousi, Mahdieh Abbasalizad Farhangi, Abnoos Mokhtari Ardekani, Faria Jafarzadeh

**Affiliations:** 1 Tabriz Health Services Management Research Center, Tabriz University of Medical Sciences, Tabriz, Iran; 2 Department of Cardiovascular Medicine, Assistant Professor of Cardiology, Lorestan University of Medical Sciences, Khorramabad, Iran; 3 Razavi Cancer Research Center, Razavi Hospital, Imam Reza International University, Mashhad, Iran; 4 Department of Nutrition in Community, Faculty of Nutrition, Tabriz University of Medical Sciences, Tabriz, Iran; 5 Endocrinology and Metabolism Research Center, Institute of Basic and Clinical Physiology Science, & Physiology Research Center, Kerman University of Medical Sciences, Kerman, Iran; 6 Assistant Professor of Endocrinology & Metabolism, Department of Internal Medicine, School of Medicine, North Khorasan University of Medical Sciences, Bojnourd, Iran

**Keywords:** Dietary antioxidant quality score, Cardio-metabolic diseases, Obesity, Overweight, Blood pressure, Metabolic profile, Glycemic markers, Metabolic syndrome

## Abstract

**Background:**

Oxidative stress (OS) is associated with a variety of non-communicable diseases, including MetS, diabetes mellitus, metabolic syndrome, and cardiovascular disease through increased production of reactive oxygen species (ROS) and impairment of antioxidant defense mechanisms. Antioxidants can protect cells against free radical damage, so it seems important to determine the relationship between the quality of dietary antioxidants intake and chronic diseases. The Dietary Antioxidant Quality Score (DAQS) is obtained by adding the daily intake of known dietary vitamins and minerals, including selenium, zinc, vitamin A, vitamin C, and vitamin E, compared to the recommended daily intake (RDI). Therefore, this study aims to determine the relationship between DAQS, serum lipids, markers of glucose homeostasis, blood pressure and anthropometric features among obese adults.

**Methods:**

In the present cross-sectional study, 338 individuals who were obese (BMI ≥ 30 kg/m^2^) aged 20-50 years were recruited from Tabriz and Tehran, Iran. A validated semi-quantitative Food Frequency Questionnaire (FFQ) with 168 food items was used to quantify dietary consumption; accordingly, DAQS was computed. Blood biomarkers were measured using enzyme-linked immunosorbent assay (ELISA) kits. A standard mercury sphygmomanometer was used to assess blood pressure, and bioelectrical impedance analysis (BIA) was performed to determine body composition. The association between the DAQS tertiles and biochemical variables was investigated using multinomial logistic regression.

**Results:**

Participants in the highest tertile of DAQS have a lower diastolic blood pressure (DBP) values in all of the adjusted models [odds ratio (OR) = 0.920; confidence interval (CI)= 0.852-0.993, *P*-value = 0.03] in the analysis of co-variance (ANCOVA) model. Similarly, subjects at the second tertile of DAQS had lower DBP compared with the first tertile in age and sex-adjusted model [OR= 0.937; CI= 0.882-0.997]. There was no statistically significant difference for other metabolic parameters in different DAQS tertiles.

**Conclusion:**

According to our findings, higher DAQS was associated with lower DBP among obese adults with obesity in two major cities of Iran (Tehran and Tabriz). Other studies with interventional design are needed to better elucidate these associations and underlying mechanisms.

## Introduction

Cardiometabolic diseases (CMD) refers to a group of metabolic abnormalities that increase the risk of developing type II diabetes mellitus (T_2_DM), systemic hypertension, central obesity, and dyslipidemia [1, 2]. Over the past 20 years, metabolic syndrome (MetS) and cardiovascular disorders have become more prevalent worldwide [3–5]. MetS prevalence varies from 8% in India to 24% in the United States, and from 7% in France to 43% in Iran, respectively [6]. This rise in cardio-metabolic illnesses imposes a major cost on individuals, healthcare organizations, and society in general [7]. A growing number of research have been conducted to investigate the impact of inflammation and oxidative stress (OS) on a variety of chronic conditions, including MetS, T_2_DM, and cardiovascular disease [8–15]. Most CMDs have enhanced reactive oxygen species (ROS) generation and impaired antioxidant defense mechanisms [16]. The major cause of endothelial dysfunction, which leads to vascular damage in both metabolic and atherosclerotic disorders, is an imbalance between the synthesis of reactive ROS and antioxidant defense mechanisms [17]. Antioxidants, which protect cells from free radical damage, include enzymes such as superoxide dismutase, glutathione reductase, glutathione peroxidase, catalase, and minerals such as selenium (Se) and zinc (Zn), as well as nonenzymatic or nutrient-derived antioxidants such as vitamins A, C, and E [18]. Vitamins A and C are effective free radical scavengers, while vitamin E defends against lipid peroxidation [19]. Human selenoproteins are a group of 25 selenium-containing proteins whose synthesis requires the insertion of a selenium-containing homolog of cysteine, the main function of multiple selenoproteins, such as glutathione peroxidase (GPX), thioredoxin reductase (TrxR), and iodothyronine deiodinases (IDD), is to act as important intra [20, 21]. Among other minerals, zinc is an inhibitor of NADPH oxidase, which leads to a decrease in ROS production. Furthermore, zinc is a cofactor of superoxide dismutase (SOD) and also promotes the formation of metallothionein, which is high in cysteine and an effective OH scavenger [22]. It is thought that analyzing the antioxidant quality of an individual's diet rather than a particular antioxidant vitamin or mineral might provide a more comprehensive picture of the relationship between dietary antioxidants and health outcomes. Hence, Dietary Antioxidant Quality Score (DAQS) is used, which analyses antioxidant-nutrient intake by adding the daily intake of specific vitamins and minerals proved to function as dietary antioxidants: selenium, zinc, vitamin A, vitamin C, and vitamin E and provides a determined amount-based score compared to the FDA-recommended quantity [23–25]. DAQS was positively associated with anthropometric parameters such as waist circumference (WC) and waist to height ratio (WHtR) in underweight and normal-weight in a nationwide cross-sectional research that was conducted among 4270 children and adolescents aged 6 - 18 years [26]. Also Shahavandi et al. [27] found that adherence to DAQS showed a significant decrease for systolic blood pressure among 270 apparently healthy adults. Emerging new research suggests that designing dietary strategies based on bioactive antioxidants can prevent metabolic disorders [28–30]. For this reason, we hypothesized that a higher quality of dietary antioxidant score may be associated with a favorable cardio-metabolic profile such as blood pressure, glycemic markers and lipid profile among adult individuals with obesity who are apparently metabolically healthy from two metropolises of Iran, Tabriz and Tehran.

## Method and materials

### Study design and participants

We conducted the current cross-sectional study among both male and female adults with obesity in Tabriz and Tehran, Iran who participated in two previous projects [31, 32] that was conducted during the last four years from 2019 to 2022. Inclusion criteria were the age range of 20–50 years old and BMI≥30 kg/m^2^ and exclusion criteria were being pregnant, breastfeeding, menopausal, CVD, cancer, any disorders related to hepatic and kidney function, having recent bariatric surgery, DM, and taking any medications that make changes in weight or metabolic metabolism. Study participants were recruited by public announcements. Full-informed approved written consent form was taken from all of the subjects and the proposal of this study was approved by the ethics committee of Tabriz University of Medical Sciences, Tabriz, Iran (registration code: IR.TBZMED.REC.1398.460).

### Anthropometric assessments

A wall-mounted stadiometer and a Seca scale (Seca Co., Hamburg, Germany) were used to evaluate the subjects' height and weight to the nearest 0.5 cm and 0.1 kg, respectively. Device Tanita, BC-418 MA (Tokyo, Japan) used the bioelectrical impedance analysis (BIA) technique to determine body composition. The body fat percentage, fat mass (FM), fat free mass (FFM), and predicted muscle mass are all calculated by this instrument. Hip circumference (HC) was determined over the broadest region of the buttocks and was recorded to the closest 0.1 cm, while waist circumference (WC) was measured at the midway between the lower costal border and the iliac crest using a tape measure.

### Dietary assessments and calculation of DAQS

At this study, we collected dietary intakes using a validated semi-quantitative Food Frequency Questionnaire (FFQ), adapted for Iranian population [33]. Participants were asked to record all the food and beverages they consumed on a daily, weekly, monthly, or yearly basis, using the Iranian household manual's basic recommendations for portion sizes, cooking yields, and edible food quantities. The stated frequency for each food item was changed to daily consumption while converted to gram. Vitamins and minerals with antioxidant effects, such as selenium, zinc, vitamin A, vitamin C, and vitamin E, were used to calculate the DAQS [34, 35]. The daily nutrient intake of participants was compared to the recommended daily intake (RDI) [36], then a value of 0 or 1 was assigned to each of the five antioxidants after their consumption was evaluated. Accordingly, when the intake of five components decreased below 2/3 of the RDI, a value of 0 was applied as well as a score of 1 was given when the intake exceeded 2/3 of the RDI. As a result, the overall DAQS ranged between 0 which indicated lowest antioxidant quality and 5 that shows highest antioxidant quality [37, 38].

### Sociodemographic data

In order to determine the socioeconomic status (SES) score, we gathered sociodemographic data via a questionnaire, including sex, age, smoking status, educational attainment, marital status, employment, and family size. SES was calculated by taking into account factors including educational attainment, job status, home ownership, and family size. A categorical variable, education, was assessed by the highest level of educational attainment, this variable was measured using a Likert scale with five possible values: 0 for illiteracy, 1 for less than a diploma, 2 for a diploma and an associate degree, 3 for a bachelor's degree, 4 for a master's degree, and 5 for higher education. Four categories were used to group the occupations of the female participants, including 0 for housewife, 1 for employee, 3 for student, 4 for self-employed, and 5 for others. The classification of the male subjects' occupations was as follows: self-employed: 5, farmer, and farm worker: 4, others: 3, workers: 2 and unemployed: 1. Also, participants were divided into three family size groups: under 3, 4-5, and over 6 persons with scores of 1, 2, and 3, respectively. Additionally, if they were a renter or a landlord, they received ratings of 1 and 2, respectively. Then, for the total SES score, each participant was given a score between 1 and 15. Participants' level of physical activity was evaluated using a shorter version of the International Physical Activity Questionnaire (IPAQ) [39, 40]. The frequency of depression, anxiety, and stress symptoms during the previous weeks was assessed using the Persian version of the DASS-21 [41]. Each of the seven questions on this questionnaire, age, marital status, educational attainment, nursing experience, work unit, shift work, and work hours per week—assesses a subject's sociodemographic characteristics. The questions are graded on a Likert scale with a maximum score of three and a minimum score of zero. The score may range between 0 and 21 on each scale. In the morning after a fast, the condition of the appetite was evaluated using a visual analogue scale (VAS) [42, 43]. The words "I'm not at all hungry" and "I have not been so hungry" were placed at the opposing ends of a 100-mm line to represent the two extremes of the VAS. This questionnaire inquired about hunger, satiety, fullness, desires for sweet, salty, and fatty meals, as well as anticipated future dietary consumption.

### Measurement of blood biomarkers

Blood pressure was measured with a standard mercury sphygmomanometer (Riester, Diplomat 1002, Jungingen, Germany) twice in same arm after at least 15 minutes of rest, and then the average of the two measurements reported. To measure blood routine, between 8:00 and 10:00 A.m. following fasting state 10 ml of the participant’s peripheral blood sample was collected into tubes that containing 0.1% ethylenediaminetetraacetic acid (EDTA) then serum and plasma samples separated using a centrifuge at 4500 rpm for 10 minutes. Until analysis, aliquots were frozen at −70◦C. Commercial kits were used to assess the serum levels of total cholesterol (using cholesterol oxidase Phenol 4-aminoantipyrine peroxidase method, Pars Azmoon, Cat No. 1500010, Iran), triglycerides (using glycerol-3-phosphate oxidase phenol 4-aminoantipyrine peroxidase method, Pars Azmoon, Cat No. 1500032, Iran), high-density lipoprotein cholesterol (by direct method, Pars Azmoon, Cat No. 1500012, Iran), and fasting blood sugar (using glucose oxidase, phenol, 4-amino antipyrine peroxidase method, Pars Azmoon, Cat No. 1500017, Iran). In addition, the low-density lipoprotein cholesterol level was calculated by the Friedewald equation [44]. Furthermore, enzyme-linked immunosorbent assay (ELISA) kits were used to measure blood insulin levels (Bioassay Technology Laboratory, Shanghai Korean Biotech, Shanghai City, Cat No. E0010Hu, China). The Homeostasis Model Assessment of Insulin Resistance (HOMA-IR) was calculated by following formula: fasting insulin (IU/ml)* fasting glucose (mmol/l) /22.5 [45], and the Quantitative Insulin Sensitivity Check Index (QUICKI) was calculated using the following formula: 1/[log fasting insulin (U/mL) + log glucose (mmol/L) [46, 47].

### Statistical analysis

Utilizing the Statistical Package for Social Sciences, the data were analyzed (SPSS, version 21.0; SPSS Inc, Chicago IL). The normality of the variables was assessed using histogram charts and the Kolmogorov-Smirnov test. The distribution was reported as a mean (SD) for normally distributed quantitative data and as a frequency (%) for normally distributed qualitative data. The Chi-square test and one-way analysis of variance (ANOVA) were used, respectively, to analyze the differences in discrete and continuous variables across DAQS quartiles. Multinomial logistic regression was used to investigate the relationship between the DAQS tertiles and biochemical variables, estimating odds ratios (ORs) and 95% confidence intervals (CIs) for the existence of cardio-metabolic risk factors among the DAQS tertiles in three multivariable-adjusted and un-adjusted models.

## Results

A total of 338 adults (with mean BMI=32.62 ±4.80 kg/m^2^) aged 40.78±9.23 years old of whom 41.79% were men were in the current cross-sectional study and the mean DAQS was 4.42±0.87. Table 1 represents the general demographic characteristics of the participants according to DAQS tertiles. Distribution of the study population in terms of weight, height, anthropometric measurements, and body composition across tertiles of DAQS was not significantly different (*P* >0.05). Also, no significant differences were seen between DAQS tertiles and DASS, appetite, BMR and physical activity (*P* > 0.05). ANCOVA comparisons after adjustment for potential confounders including age, gender, BMI, physical activity and energy intake found no significant difference for biochemical parameters such as SBP, DBP, FBS, TC, TG, HDL, LDL, Insulin, HOMA-IR, QUICKI (*P* >0.05). Dietary intakes of DAQS components among DAQS tertiles (Table 2) show a significant direct association between tertiles in subjects for vitamins A, C, and E, zinc and selenium (*P*<0.001). The selected dietary intake of study subjects across tertiles of DAQS is shown in Table 3. We observed a significant association between tertiles in participants for vegetables, grains, energy, fiber, carbohydrate and fat percentage, cholesterol, SFA, MUFA and PUFA (*P* < 0.05). The crude and adjusted multivariate odds ratio for cardio-metabolic risk factors across tertiles of DAQS is indicated in Table 4. In the second tertile of DAQS, DBP levels in model 2 had a significant decrease compared with first tertile (OR=0.937, *P*=0.04). Participants at the third tertile of DAQS had lower DBP in crude model (OR= 0.940, *P*=0.01), after adjustment for age and sex (OR= 0.923, *P*=0.003) as well as after adjustment for age, BMI, sex, physical activity, SES and energy intake (OR= 0.920, *P*=0.03). However, we failed to find any significant association between tertiles of DAQS and other cardio-metabolic risk factors either before or after adjustment for covariates in three models (*P*>0.05).

**Table 1:** General demographic characteristics of study participants by tertiles of dietary antioxidant quality score

Variable	**Tertiles of DAQS**						
	**1**^**st**^ ^**DAQS=1-3**^ ^**(*N*=111)**^		**2**^**nd**^ ^**DAQS=4**^ ^**(*N*=113)**^		**3**^**rd**^ ^**DAQS=5**^ ^**(*N*=114)**^		***P*** * value
	**Mean**	**SD**	**Mean**	**SD**	**Mean**	**SD**	
Age (y)	39.89	9.36	41.68	7.70	40.47	9.60	0.52*
Gender (% Male)	66.10	47.77	60.90	49.16	53.5	49.93	0.06**
Weight	89.61	12.33	93.33	15.34	92.36	14.62	0.33*
Height	169.05	9.94	168.57	11.19	167.37	9.46	0.43*
BMI (kg/m^2^)	31.43	4.16	32.91	5.01	32.93	4.89	0.10*
WC (cm)	105.50	7.93	107.72	9.15	106.68	10.13	0.44*
FM (%)	32.10	7.48	34.94	10.62	33.90	9.02	0.42*
FFM (%)	61.17	11.52	61.54	12.20	62.79	12.69	0.74*
WHR	0.93	0.07	0.94	0.07	0.93	0.08	0.89*
SES	9.66	2.74	9.85	2.65	10.09	2.41	0.66*
DASS	21.62	11.25	19.95	11.20	19.83	11.80	0.73*
Appetite	31.75	8.34	33.00	9.51	34.28	8.89	0.33*
BMR (Kcal)	7756.53	1320.84	7825.87	1431.66	7893.80	1754.27	0.91*
PA (min/week)	1881.03	3286.66	2024.30	2768.26	2284.47	3357.98	0.78*
SBP (mmHg)	122.16	13.27	123.74	13.12	122.59	17.80	0.84***
DBP (mmHg)	83.77	10.01	82.01	8.87	80.98	12.90	0.27***
FBS (mg/dl)	89.29	9.97	95.09	30.38	92.96	16.46	0.25***
TC (mg/dl)	193.51	36.00	186.87	34.81	192.81	37.75	0.47***
TG (mg/dl)	137.89	70.31	154.20	105.04	152.79	94.61	0.54***
HDL (mg/dl)	44.53	8.82	42.51	9.92	43.63	9.57	0.49***
LDL (mg/dl)	124.72	29.92	117.99	31.42	124.97	32.74	0.28***
Insulin (mIU/l)	17.93	23.93	17.92	12.13	15.17	10.11	0.32***
HOMA-IR	3.97	5.29	4.27	3.16	3.56	2.57	0.38***
QUICKI	0.33	0.04	0.32	0.03	0.33	0.04	0.14***

*DAQS* Dietary antioxidant quality score, *N* Number of participants in each tertile of DAQS, *BMI* Body mass index, *WC* Waist Circumference, *FM* Fat Mass, *FFM* Fat Free Mass, *WHR* Waist-to-hip ratio, *BMR* Basal Metabolic Rate, *SES* socio-economic status, *DASS* Depression, anxiety, and stress symptoms, *PA* Physical activity, *SBP* Systolic Blood Pressure, *DBP* Diastolic Blood Pressure, *TC* Total Cholesterol, *TG* Triglyceride, *HDL-C* High Density Lipoprotein Cholesterol, *LDL-C* Low Density Lipoprotein Cholesterol, *HOMA-IR* Homeostatic Model Assessment for Insulin Resistance, *QUICKI* Quantitative Insulin sensitivity Check Index; all data are mean (±SD) except for gender, that is presented as the number and percent of males in each group

P*values derived from One-Way ANOVA with Tukey’s post-hoc comparisons

^**^P values derived from chi-squared test

P*** values derived from ANCOVA comparisons after adjustment for confounders (age, gender, BMI, PA and kcal)

**Table 2:** Dietary intakes of DAQs components according to tertiles of dietary antioxidant quality score

Variable	**Tertiles of DAQS**						
	**1**^**st**^ ^**DAQS=1-3**^ ^**(*N*=111)**^		**2**^**nd**^ ^**DAQS=4**^ ^**(*N*=113)**^		**3**^**rd**^ ^**DAQS=5**^ ^**(*N*=114)**^		***P*** * **value**
	**Mean**	**SD**	**Mean**	**SD**	**Mean**	**SD**	
Vitamin A (μg/d)	362.48	129.61	608.20	315.77	1139.46	738.82	**<0.001**
Vitamin C (mg/d)	95.27	64.42	180.86	97.92	299.19	194.31	**<0.001**
Vitamin E (mg/d)	9.30	4.27	12.74	5.43	19.20	8.37	**<0.001**
Zinc (mg/d)	9.67	3.49	12.83	3.94	16.78	7.40	**<0.001**
Selenium (μg/d)	112.74	50.16	147.15	59.28	164.02	61.70	**<0.001**

The comparison of dietary antioxidant quality score (DAQS) and its components by dietary DAQS tertiles. N, number of participants in each tertile of DAQS. All data are represented as mean (±SD) *P-values are derived from one way ANOVA test

**Table 3:** Food groups intake of study participants by tertiles of dietary antioxidant quality score

Variable	**Tertiles of DAQS**							
	**1**^**st**^ ^**DAQS=1-3**^ ^**(*N*=111)**^		**2**^**nd**^ ^**DAQS=4**^ ^**(*N*=113)**^		**3**^**rd**^ ^**DAQS=5**^ ^**(*N*=114)**^		***P*** * **value**	***P*** ** **value**
	**Mean**	**SD**	**Mean**	**SD**	**Mean**	**SD**		
Fruits (g/d)	2.47	3.40	3.63	1.90	4.81	3.18	<0.001	0.72
Vegetables (g/d)	1.91	0.92	3.29	1.88	4.58	2.28	<0.001	**0.01**
MFP (g/d)	2.14	1.01	2.45	1.15	3.83	1.84	<0.001	0.07
Beans	0.44	0.32	0.59	0.31	0.88	0.84	0.003	0.65
Dairy (g/d)	1.22	0.78	1.97	1.11	2.35	1.39	<0.001	0.12
Grains (g/d)	11.32	6.12	13.62	6.47	15.28	7.11	0.01	**0.001**
Energy (kcal/d)	2029.73	673.15	2636.08	733.91	3405.77	1079.30	<0.001	**<0.001**
Fiber (g/d)	51.26	32.42	66.03	38.77	79.91	46.41	<0.001	**0.02**
CHO (%)	60.17	6.64	60.20	6.03	56.60	6.95	0.002	**0.005**
Protein (%)	12.99	2.14	13.04	1.83	13.01	1.99	1.00	0.08
Fat (%)	29.15	6.44	29.33	5.84	33.14	7.02	<0.001	**0.01**
Cholesterol (mg/d)	196.00	119.13	253.62	140.62	337.05	219.64	<0.001	**0.02**
SFA (g/d)	18.29	7.04	25.48	12.31	33.55	15.46	<0.001	**<0.001**
MUFA (g/d)	20.93	9.84	27.79	12.70	38.34	17.03	<0.001	**0.04**
PUFA (g/d)	14.68	8.33	17.81	8.49	26.25	14.16	<0.001	**<0.001**

*N* Number of participants in each tertile of DAQS, *MFP* Meat, fish and poultry, *CHO* Carbohydrate, *SFA* Saturated fatty acids, *MUFA* Mono-unsaturated fatty acids, *PUFA* Polyunsaturated fatty acids

All data are mean (±SD)

P* values derived from unadjusted ANCOVA

P** values derived from ANCOVA after, adjustment for confounders (age, gender, BMI, PA and energy intake)

**Table 4:** Biochemical variables of study participants by tertiles of dietary antioxidant quality score

**Variable**		**Tertiles of DAQS**				
		**1**^**st**^ ^**DAQS=1-3**^ ^**(*N*=111)**^	**2**^**nd**^ ^**DAQS=4**^ ^**(*N*=113)**^		**3**^**rd**^ ^**DAQS=5**^ ^**(*N*=114)**^	
			OR(CI)	*P*-value	OR(CI)	*P*-value
SBP (mmHg)	Model I	**1** **REF**	1.036(0.993-1.081)	0.10	1.029(0.992-1.067)	0.12
	Model II		1.042(0.995-1.090)	0.08	1.039(0.999-1.081)	0.06
	Model III		1.037(0.971-1.108)	0.27	1.027(0.964-1.094)	0.40
DBP (mmHg)	Model I	**1** **REF**	0.954(0.901-1.010)	0.11	0.940(0.894-0.987)	**0.01**
	Model II		0.937(0.882-0.997)	**0.04**	0.923(0.874-0.974)	**0.003**
	Model III		0.944 (0874-1.020)	0.14	0.920(0.852-0.993)	**0.03**
FBS (mg/dl)	Model I	**1** **REF**	1.012(0.967-1.060)	0.60	1.007(0.967-1.050)	0.72
	Model II		1.011(0.964 1.060)	0.65	1.011(0.968-1.055)	0.62
	Model III		0.999(0.911-1.095)	0.98	1.018(0.930-1.114)	0.70
TC (mg/dl)	Model I	**1** **REF**	0.973(0.916-1.034)	0.38	0.956(0.907-1.007)	0.09
	Model II		0.973(0.917-1.031)	0.35	0.954(0.906-1.004)	0.07
	Model III		0.996(0.977-1.016)	0.71	1.015(0.997-1.033)	0.11
TG (mg/dl)	Model I	**1** **REF**	1.006(0.994-1.019)	0.32	1.009(0.999-1.020)	0.09
	Model II		1.007(0.994-1.019)	0.30	1.010(0.999-1.021)	0.07
	Model III		1.000(0.988-1.012)	0.98	0.998(0.987-1.010)	0.76
HDL (mg/dl)	Model I	**1** **REF**	1.019(0.943-1.101)	0.64	1.043(0.976-1.115)	0.21
	Model II		1.011(0.935-1.093)	0.79	1.030(0.962-1.102)	0.40
	Model III		0.979(0.909-1.055)	0.57	0.977(0.912-1.048)	0.52
LDL (mg/dl)	Model I	**1** **REF**	1.019(0.958-1.083)	0.55	1.047(0.993-1.104)	0.09
	Model II		1.019(0.960-1.081)	0.53	1.049(0.997-1.105)	0.07
	Model III		1.018(0.964-1.078)	0.50	1.049(0.996-1.107)	0.08
Insulin (mIU/l)	Model I	**1** **REF**	0.967(0.798-1.171)	0.73	0.942(0.789-1.124)	0.51
	Model II		0.981(0.805-1.196)	0.85	0.971(0.808-1.166)	0.75
	Model III		0.863(0.523-1.425)	0.57	0.971(0.593-1.590)	0.91
HOMA-IR	Model I	**1** **REF**	1.047(0.438-2.504)	0.92	1.172(0.532-2.580)	0.69
	Model II		0.943(0.388-2.292)	0.90	0.996(0.446-2.223)	0.99
	Model III		1.450(0.249-8.446)	0.68	0.937(0.165-5.341)	0.94
QUICKI	Model I	**1** **REF**	1.120E-7(4.088E-16-30.658)	0.11	0.000(1.407E-10-1189.970)	0.30
	Model II		1.031E-8(1.817E-17-5.852)	0.07	0.000(1.846E-11-1223.025)	0.28
	Model III		1.553E-11(1.326E-33-1.818E+11)	0.34	3.657E-8(1.237E-28-1.081E+13)	0.48

The multivariate multinomial logistic regression was used for the estimation of ORs and confidence interval (CI). Model I: crude, Model II: adjusted for age and sex, Model III: adjusted for age, BMI, sex, physical activity, SES and energy intake

*DAQS* Dietary antioxidant quality score, *N* Number of participants in each tertile of DAQS, *SBP* Systolic Blood Pressure, *DBP* Diastolic Blood Pressure, *TC* Total Cholesterol, *TG* Triglyceride, *HDL-C* High Density Lipoprotein Cholesterol, *LDL-C* Low Density Lipoprotein Cholesterol, *HOMA-IR* Homeostatic Model Assessment for Insulin Resistance, *QUICKI* Quantitative Insulin Sensitivity Check Index, *OR* odds ratio, *CI* Confidence interval

## Discussion

**Fig. 1:**
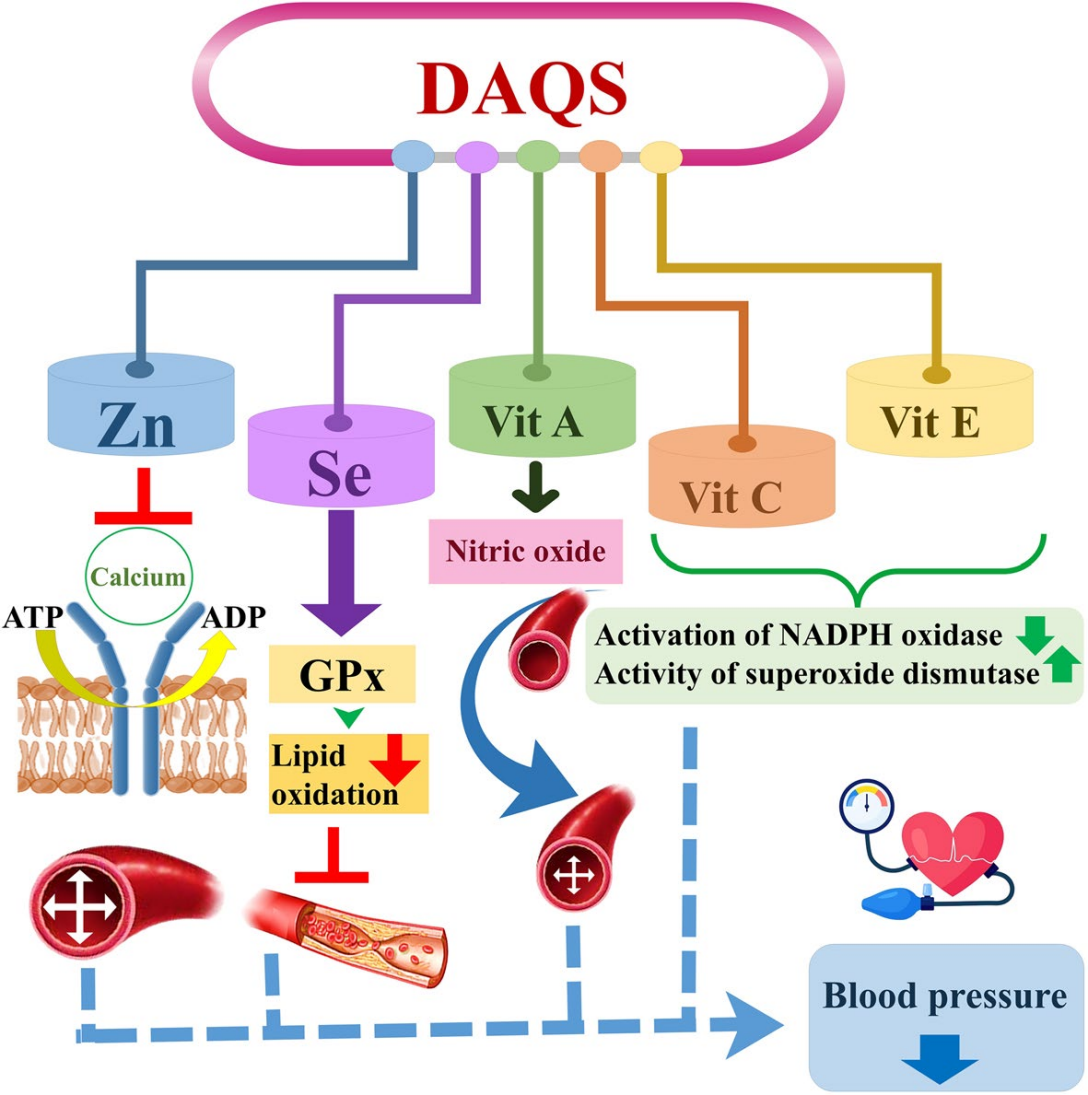
Summarized mechanistic pathways of potential effects of DAQS components on blood pressure. Abbreviations: DAQS, dietary antioxidant quality score; Zn, zinc; Se, selenium; ATP, adenosine triphosphate; ADP, adenosine diphosphate; GPx, glutathione peroxidase; Vit, vitamin

The association of selective dietary antioxidants and cardio-metabolic risk factors such as anthropometric features, blood pressure, lipid profile and markers of glucose homeostasis has been identified in several studies [48–52]. This method, which focuses on the impact of a few specific antioxidants on health outcomes, lacks a lot of information regarding the complicated or cumulative relationships and interactions between antioxidants in foods. Therefore, it seems necessary to study the relationship between the total intake of antioxidant vitamins and minerals and the quality of their intake in the diet with health risk factors, such as lipid profile, glycemic markers, and blood pressure. This cross-sectional study examined the association between dietary antioxidant quality score (DAQS) of several vitamins and minerals that have been reported to have antioxidant functions including selenium, zinc, vitamin A, vitamin C, and vitamin E and cardio-metabolic risk factors and MetS components in a sample of apparently healthy Iranian adults with obesity. Our findings indicate a non-significant relationship between DAQS and cardio-metabolic risk factors by ANCOVA comparisons. However, a positive association between DAQS tertiles and the odds of decreasing DBP levels was found which means participants in the highest tertile of DAQS had a lower chance for higher DBP levels. In line with our findings, Waśkiewicz A [53] suggested a significant association between higher dietary antioxidant potential and a lower chance of hypertension among the 15,120 Polish adults. Also Maugeria et al. [54] in a cross-sectional analysis among 894 subjects found that dietary antioxidants such as vitamin A, vitamin C, vitamin E, zinc, selenium, and carotenoids inversely correlated with several cardiovascular risk factors including WHR, body fat mass, systolic and diastolic blood pressure, creatinine and total cholesterol/HDL ratio . Nevertheless Chen J et al. [55] observed that two antioxidant vitamins of serum, vitamins A and E, were linked to a 43% and 18% increased risk of hypertension, respectively. A cross-sectional research by Shahavandi et al. which included 270 adult individuals aged 18-45 years old from Tehran, Iran, although revealed that participants in higher dietary antioxidant quality score tertiles had a significant lower SBP levels, it is indicated that the risk of MetS and its components such as elevated TG and BP, higher levels of FBS, Low HDL and high WC not changed among tertiles of DAQS [27]. The findings of this study support the results obtained in the present study, we also could not find a significant association between SBP, FBS, TC, TG, HDL, LDL, insulin, HOMA-IR and QUICKI among DAQS tertiles in crude model and even after two adjustment model. An explanation for these findings may be that these results were obtained in a healthy population without a major micronutrient deficiency or diagnosed with pro-antioxidant imbalance, so examining DAQS in people with metabolic diseases can reveal a significant relationship between DAQS and components of metabolic syndrome and cardio-metabolic risk factors. Each of the five antioxidants examined in this study can alone have a role in reducing blood pressure as seen in the present study. Zinc is found in numerous enzymes involved in the regulation of arterial blood pressure, including angiotensin-converting enzyme and neutral endopeptidases [56, 57] also Zn inhibits the adenosine triphosphate dependent calcium pump, which releases calcium from cells and increases calcium levels in vascular walls. According to the study of Kin J, dietary zinc consumption is highly related to SBP in obese Korean women [58]. However, a prospective study that follows up 1652 men without a known history of hypertension for almost 24 years concluded a positive association between higher serum zinc concentration and incident hypertension. Selenium is an essential component of glutathione peroxidase (GPx), an enzyme which reduces lipid oxidation and the development of atherosclerotic plaques [59]. Selenium deficiency could be a risk factor for higher blood pressure levels [59]. However, systematic review study by Kuruppu D et al. [60] revealed that there is no conclusive evidence establishing a link between selenium and hypertension. Also a large scale of data suggests that vitamins with antioxidant characteristics, such vitamins C and E, can reduce blood pressure [61–64]. Enhanced endothelium-dependent vasodilation, amelioration of vascular structural alterations, reduced activation of NADPH oxidase, and enhanced activity of superoxide dismutase are mechanistic pathways that contribute to the blood pressure-lowering effects of vitamins C and E [65]. Vitamin A has been shown to have a major role in oxidative stress, and endothelial function [66, 67]. A prospective cohort study which included 12,245 individuals found that the risk of hypertension was significantly increased in the lowest quartile of vitamin A intake levels [68]. Nitric oxide (NO) is crucial for controlling blood pressure and flow because to its vasodilatory actions [69], NO pathways were shown to be affected by vitamin A, which may contribute to endothelial function [66, 70]. Some of the mechanistic pathways involved in the association between DAQS and blood pressure is illustrated in Figure 1. In our current study, people in higher DAQS tertiles had higher consumption of foods with higher nutritional and antioxidant value such as vegetables and fiber. However, higher intake of saturated and unsaturated fatty acids and cholesterol was also observed. This study should be considered within the context of its possible limitations. Although we obtained data from a well-designed population-based study, our cross-sectional design method it is difficult to understand the mechanistic pathway and draw conclusions about causality. In addition, our results are representative of a population in Tabriz and Tehran cities of Iran. Consequently, it is advised to use caution when generalizing the findings to other regions. Using FFQ can cause bias due to memory-based and long-term food intake recording. Nonetheless, we gathered dietary intakes of participants using a valid and reliable FFQ which is adapted for our target sample size. Moreover, we performed multiple adjusting models to modify several confounding factors to increase the reliability of findings.

## Conclusions

According to the findings of the present study, higher DAQS was associated with lower diastolic blood pressure but there is no significant relationship between other cardiovascular risk factors including serum lipids and lipoproteins, glycemic markers, and insulin resistance biomarkers among obese individuals in Tabriz and Tehran, Iran.

## Data Availability

The datasets generated and/or analyzed during the current study are not publicly available due to privacy and ethical considerations, but can be available from the corresponding author on reasonable request.
